# Epigenetic silencing of KLF2 by long non-coding RNA SNHG1 inhibits periodontal ligament stem cell osteogenesis differentiation

**DOI:** 10.1186/s13287-020-01953-8

**Published:** 2020-10-07

**Authors:** Zhaobao Li, Xiangjun Guo, Shuainan Wu

**Affiliations:** Department of Stomatology Clinic, Cangzhou Central Hospital, Cangzhou, 061000 Hebei China

**Keywords:** SNHG1, EZH2, KLF2, PDLSCs, Osteogenic differentiation

## Abstract

**Background:**

Exploring the effects of lncRNA SNHG1 in the process of osteogenic differentiation of periodontal ligament stem cells (PDLSCs) would provide novel therapeutic strategies for tissue regeneration.

**Methods:**

Loss-of-function and gain-of-function assays were induced by lentivirus. The osteogenic differentiation of PDLSCs were assessed by ALP staining and Alizarin Red staining as well as the mRNA and protein levels of osteogenic marker genes osterix, osteocalcin, and alkaline phosphatase through qRT-PCR and western blot. RNA immunoprecipitation assay and chromatin immunoprecipitation assays were performed to uncover the interaction between SNHG1 and EZH2.

**Results:**

Our analysis revealed that SNHG1 was downregulated and KLF2 was upregulated during the osteogenic induction differentiation of PDLSCs. SNHG1 inhibited while KLF2 promoted osteogenic differentiation of PDLSCs. SNHG1 directly interact with the histone methyltransferase enhancer of the zeste homolog 2 (EZH2) and modulate the histone methylation of promoter of Kruppel-like factor 2 (KLF2) and altered the progress osteogenic differentiation of PDLSCs.

**Conclusions:**

Taken together, SNHG1 inhibited the osteogenic differentiation of PDLSCs through EZH2-mediated H3K27me3 methylation of KLF2 promotor and provided a novel class of therapeutic targets for regenerate dental tissues.

## Background

Human periodontal mesenchymal stem cells (PDLSCs) are a group of mesenchymal stem cells which are isolated from periodontal ligament tissue with multiple differentiation capability and show a promising potential of being used to regenerate supporting tissues for the patients [1–3]. Periodontitis is a highly prevalent chronic inflammatory bone disease and could destroy the periodontal structures, including alveolar bone, periodontal ligament, and root cementum, which eventually lead to tooth-loss [4]. Therefore, better understanding of the mechanism that governs PDLSCs osteogenic differentiation would greatly be beneficial to the development of novel therapeutic strategies for tissue regeneration.

Long noncoding RNAs (lncRNAs), with longer than 200 nucleotides in length, have recently been noticed as the function on malignant processes, such as carcinogenesis [5–7] and periodontitis [8–10]. LncRNA ANCR was report to inhibit osteogenic differentiation of PDLSCs via sponging miRNA-758 [11]. Similarly, lncRNA H19 also was reported to promote osteogenic differentiation of rat ectomesenchymal stem cells through enhancing the expressions of osteogenic markers, beta-catenin, and target genes of Wnt/beta-catenin signaling pathway partly through sponging miR-22 and miR-141 [12]. LncRNA SNHG1 could inhibit osteogenic differentiation of bone marrow mesenchymal stem cells through p38 MAPK pathway [13]. In brief, lncRNA plays an important role in osteogenic differentiation of PDLSCs and our study will further investigate the effects of lncRNA SNHG1 in osteogenic differentiation of PDLSCs and explore the underlying mechanism.

LncRNAs are reported to exert their functions through different molecular mechanisms and impact the transcription of downstream factors by regulating histone acetylation or methylation as well as DNA methylation or hydroxy methylation [14–16]. EZH2 (enhancer of the zeste homolog 2) which was characterized as a histone methylase was also found to interact with lncRNAs and then impact the transcription of downstream factors [17]. For instance, LINC00114 suppressed miR-133b expression via EZH2/DNMT1-mediated methylation of its promoter region and facilitated colorectal cancer development [16]. Long noncoding RNA NEAT1 (nuclear-enriched abundant transcript 1) inhibited hepatocyte proliferation in fulminant hepatic failure via strengthened recruitment of EZH2 to the LATS2 (large tumor-suppressor kinase 2) promoter region [18]. Importantly, SNHG1 was also proved to interact with EZH2 and inhibit CDKN2B (cyclin-dependent kinase inhibitor 2B) and CDKN1A (cyclin-dependent kinase inhibitor 1A) transcription [19, 20]. However, the function of SNHG1 and EZH2 in osteogenic differentiation of PDLSCs was still less reported.

The purpose of the current study was to investigate whether lncRNA SNHG1 was mechanistically involved in the osteogenic differentiation of PDLSCs. Our results identified that SNHG1 was a regulator of PDLSC osteogenesis and found SNHG1 participates in epigenetic repression of KLF2 by interacting with EZH2 in the progress of the osteogenic differentiation of PDLSCs. Our results provided novel insights into the mechanism that underlies the osteogenic differentiation of PDLSCs and could serve as novel therapeutic strategies for regenerate dental tissues.

## Materials and methods

### Isolation and culture of primary cells

The first or the second premolar teeth from 10 patients without periodontal disease (age 18–25 years) were respectively collected for orthodontic purposes. Ten premolar teeth were pooled and used to collect the periodontal membrane tissue. This study was approved by the ethics committee of Cangzhou Central Hospital. Written informed consent was obtained from all participants. All research involving human stem cells complied with the ISSCR “Guidelines for the Conduct of Human Embryonic Stem Cell Research.”

PDLSCs were isolated, cultured, and identified as previously reported [21]. Briefly, periodontal ligament tissues were then separated from middle 1/3 of teeth roots. Tissue fragments were then digested with collagenase type I and dispase (Sigma-Aldrich) for 1 h at 37 °C. Cell suspensions were then filtered through a 70-μm cell strainer (BD Biosciences) and single cell suspension were maintained in complete α-MEM (HyClone) with 10% FBS (Invitrogen) at 37 °C with 5% CO_2_. The culture medium was changed every 3 days. Cells at passages 3–5 were used in subsequent experiments.

### Flow cytometry analysis

For immunophenotype characterization, 1 × 10^5^ PDLSCs were incubated with anti-human stem cell surface-labeled antibodies including CD73-phycoerythrin and CD90-phycoerythrin (BD Biosciences, USA), anti-hematopoietic marker CD34 antibodies (BD Biosciences, USA), and anti-endothelial cell marker CD31 antibodies (BD Biosciences, USA). All flow cytometry tests were performed on a FACSAria (BD Bioscience).

### Multiple differentiation of cells in vitro

The multiple differentiation capacities of PDLSCs were determined according to the methods previously described [22, 23]. Briefly, PDLSCs were cultured into a 6-well plate in a density of 1 × 10^6^ cell per well without inducers until 80% confluence. For osteogenic induction, osteogenic differentiation induction medium (50 μg/mL of ascorbic acid (Sigma), 10 mM of beta-glycerophosphate (Sigma), and 10 nM of dexamethasone (Sigma) diluted in 10% FBS a-MEM) was given for 2 weeks and PDLSCs were identified with alizarin red and ALP staining. For adipogenic differentiation, adipogenic medium (StemPro Adipogenesis Differentiation Kit) for 14 days and then fixed and stained with oil red O staining. For chondrogenic differentiation, 1 × 10^5^ of cells were cultured in a 24-well cell culture dish with the chondrogenic medium (StemPro Chondrogenesis Differentiation Kit). After 2 weeks of induction, the clusters were fixed with 4% paraformaldehyde, embedded in paraffin. Five-micrometer section was used for alcian blue staining (Cyagen, HUXMA-90041) for identification.

### Virus infection

Virus for SNHG1, KLF2, and EZH2 overexpression and the specific shRNA for KLF2, SNHG1, and EZH2 were prepared by GenePharma. Viral infection was conducted as Liu et al. described [24]. PDLSCs were inoculated overnight and infected with lentiviruses in the presence of polybrene (6 μg/mL; Sigma-Aldrich) for 6 h. Cells were then selected with puromycin treatment for 48 h. Resistant clones were collected and was identified via real-time RT-PCR and western blot. The sequences of shRNA are shown in supplementary Table 1.

### Alkaline phosphatase (ALP) activity and ALP staining assay

PDLSCs were seeded into 96-well plates with a density of 1 × 10^5^ cells/well. Fourteen days after induction, ALP activity was detected through the ALP assay kit (Jiancheng Technology Co.). ALP activity was determined at 405 nm using p-nitro-phenyl phosphate as a substrate. Cellular ALP was also visualized by using Alkaline Phosphatase Color Development Kit (Beyotime) according to the manufacturer’s protocol.

### Alizarin red staining and quantification

PDLSCs were transferred into 24-well plates with a density of 2 × 10^5^ cells/well. Fourteen days after induction, alizarin red staining measurement was performed by using Alizarin Red S (0.2%, Solarbio, pH = 8.3) at 24–26 °C. After washing in PBS, the matrix mineralization level was observed using an inverted microscope. To detect the concentration of calcium deposits, the Alizarin Red dye in the PDLSCs was extracted with 400 μl of 10% (w/v) cetylpyridinium chloride in 10 mM sodium phosphate solution for 10 min and then quantified on a UV-Vis spectrometer at 562 nm.

### Real-time quantitative polymerase chain reaction

Total RNAs were extract using TRIzol reagent (CW Biotech) followed the manufacturers’ instructions. Super cDNA First-Strand Synthesis Kit (CW Biotech) and SYBR Green Real-Time PCR Master Mix (Invitrogen) were used for mRNA detection. GAPDH was used as endogenous controls for mRNA detection and the relative level of gene expression was calculated based on the 2^−DDCq^ method. The primers are shown in supplementary Table 2.

### Western blot analysis

PDLSCs were lysed in RIPA buffer (Sigma-Aldrich) and western blotting was performed using SDS-PAGE and then transferred to PVDF membranes (Merck Millipore). After blocking in 5% milk, the blot was incubated with primary and secondary antibodies. All antibodies were purchased from Abcam. The enhanced chemiluminescence (ECL) reagents were added to visualize the protein band and GAPDH was taken as the loading control. The gray intensity analysis was performed using Image Pro Plus (Media Cybernetics). All antibodies were purchased form Abcam and listed in supplementary Table 3.

### RNA immunoprecipitation (RIP)-PCR assay

RIP was performed using the RIP Kit (BersinBio) with EZH2 antibody according to the manufacturer’s instructions. 1 × 10^7^ treated PDLSCs were lysed with RIP buffer. The cell lysate was then incubated with sepharose beads (Bio-Rad, Hercules, CA, USA) pre-coated with EZH2 antibody (Abcam). Immunoglobulin G antibody served as the control in this study. qRT-PCR was performed as described above to measure the levels of SNHG1 associated with EZH2. The results were normalized relative to the input control.

### Chromatin immunoprecipitation (ChIP)-PCR assay

ChIP was carried out using the Pierce Agarose ChIP kit (ThermoFisher Scientific) as described previously [20]. Briefly, 1 × 10^7^ treated PDLSCs were cross-linked with 1% formaldehyde solution for 10 min at room temperature. DNA fragments ranging from 200 to 500 bp were generated via sonication. Then, the lysates were immunoprecipitated with anti-EZH2, anti-H3K27me3, or normal rabbit IgG antibody. Immunoprecipitated DNAs were analyzed by qRT-PCR. The results were normalized relative to the input control. All antibodies were purchased form Abcam and listed in supplementary Table 3.

### Statistical analysis

The results were presented as means ± standard deviation using Graphpad Prism 6 (GraphPad Software Inc.). All the data used in study normally distributed and meet homogeneity test for variance. The collected data for comparisons were analyzed by T Student’s *t* test and one-way analysis of variance (ANOVA) for comparisons. *p* < 0.05 was considered statistically significant.

## Results

### SNHG1 and KLF2 expression in PDLSCs during the osteogenic differentiation

**Fig. 1:**
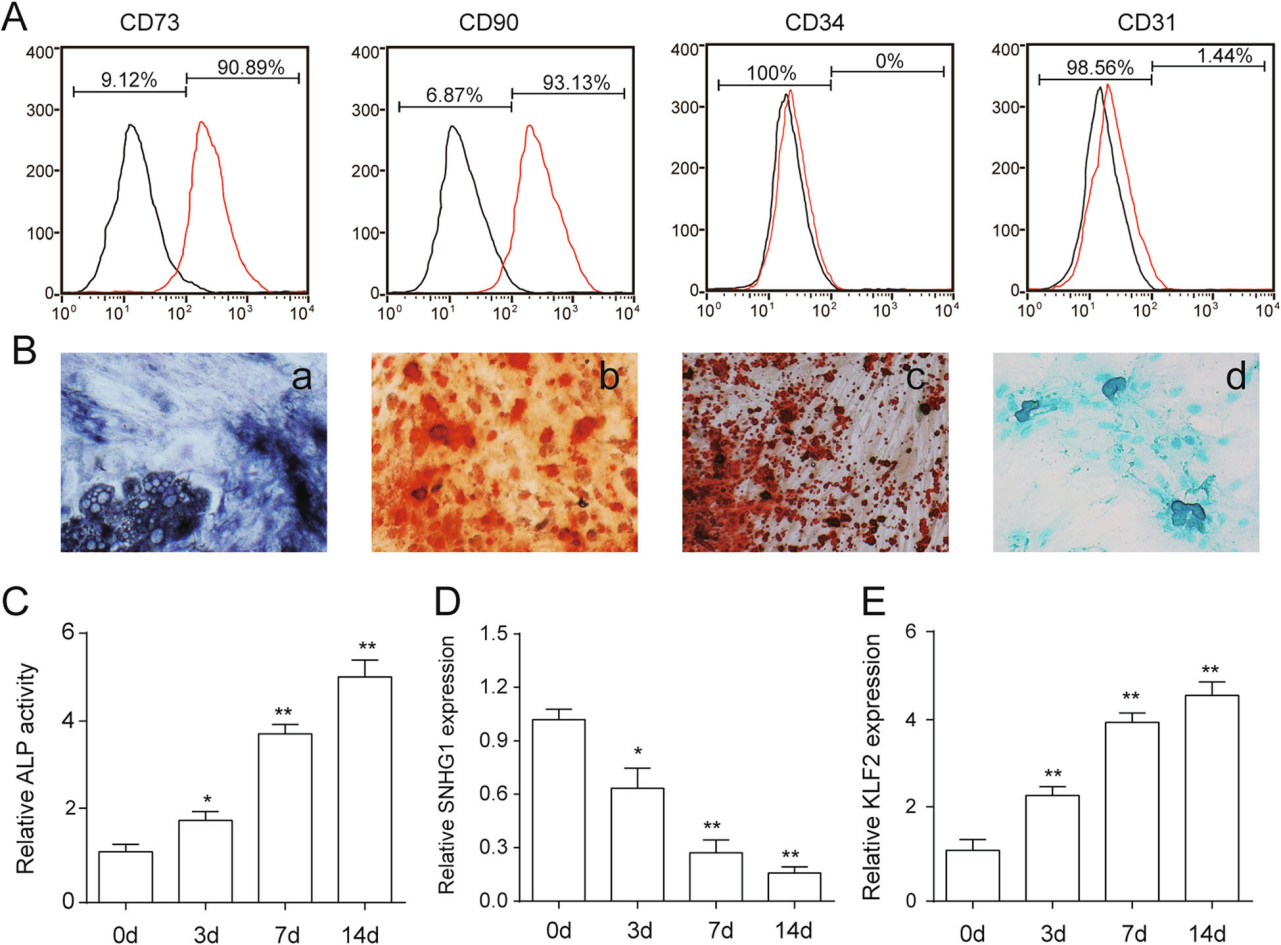
SNHG1 was downregulated and KLF2 was upregulated in osteogenic differentiated PDLSCs. **A** Flow cytometric analysis showing positive expression of CD73 and CD90 and negative expression of CD31 and CD34 in PDLSCs. **B** Multiple differentiation of PDLSCs. Osteogenic differentiation ability of PDLSCs assayed by ALP staining (a) and alizarin red staining (b). Adipogenic differentiation ability of PDLSCs assayed by oil red O staining (c). Chondrogenic differentiation ability of PDLSCs assayed by alcian blue staining (d). **C** Relative ALP activity was measured at day 3, 7, and 14. **D**, **E** The mRNA expression levels of SNHG1 (**D**) and KLF2 (**E**) were analyzed by qRT-PCR at day 3, 7, and 14, with GAPDH as a control. *p < 0.05; **p < 0.01 versus day 0

PDLSCs were successfully isolated and exhibited the positive mesenchymal stem cell surface markers, including CD73 and CD90, whereas the negative hematopoietic marker CD34 and endothelial cell marker CD31 (Fig. 1A). PDLSCs also were found to differentiate into osteogenic, chondrogenic, and adipogenic lineages after relevant induction protocols (Fig. 1B). To investigate whether SNHG1 and KLF2 are involved in regulating osteogenic differentiation, PDLSCs were cultured in osteogenic differentiation medium (OM). The activity of osteoblastic marker ALP was significantly increased after OM treatment for 3, 7, and 14 days, which confirmed the osteoblast phenotype (Fig. 1C). Next, the expression of SNHG1 and KLF2 were examined at different time points during osteoblast differentiation. As shown in Fig. 1D and E, the results suggested a gradual upregulation of the KLF2 expression and a gradual downregulation of the SNHG1 expression during the osteogenic induction differentiation of PDLSCs, indicating that SNHG1 and KLF2 might be involved in regulating the osteogenic differentiation of PDLSCs.

### SNHG1 inhibited osteogenic differentiation of PDLSCs

**Fig. 2:**
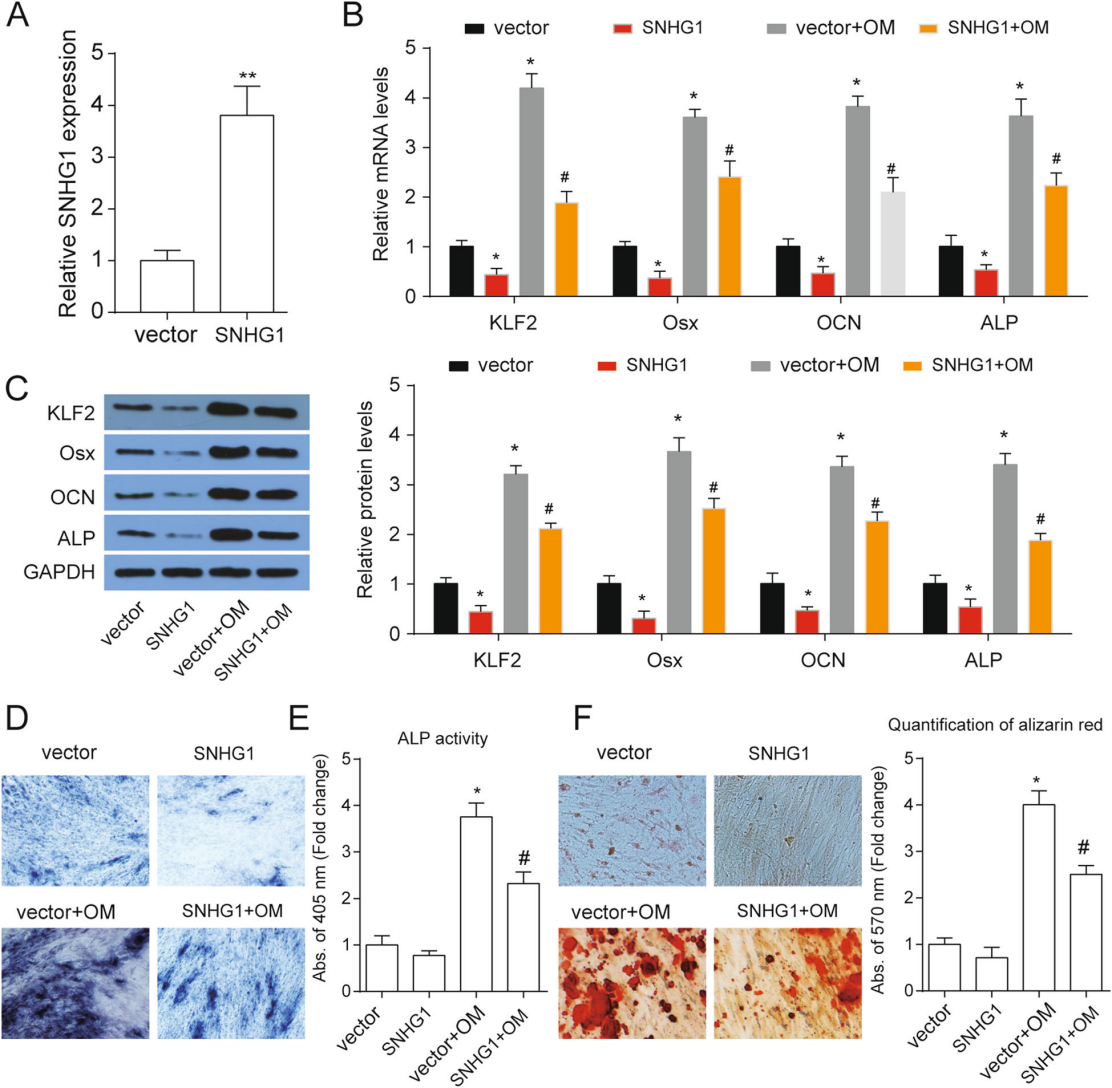
SNHG1 inhibited osteogenic differentiation of PDLSCs. **a** SNHG1 expression in PDLSCs after infected SNHG1-lentivirus. **b** The mRNA expressions of KLF2 and osteoblastic marker genes Osx, OCN, and ALP were analyzed at day 14 of osteogenic differentiation after indicated treatment with GAPDH as a control. **c** The protein levels KLF2 and osteoblastic marker genes Osx, OCN, and ALP were analyzed at day 14 of osteogenic differentiation after indicated treatment with GAPDH as a control. **d** ALP staining of PDLSCs at day 14 of osteogenic differentiation after indicated treatment. **e** ALP activity was measured at day 14 of osteogenic differentiation after indicated treatment. **f** Alizarin Red staining of PDLSCs at day 14 of osteogenic differentiation after indicated treatment and quantification was shown at right. *p < 0.05 versus vector; ^#^*p* < 0.05 versus vector+OM

To identify the biological role of SNHG1 in the regulation of PDLSCs differentiation, cells were infected with SNHG1-lentivirus to establish stably SNHG1-overexpressing PDLSCs (Fig. 2a). SNHG1 overexpression apparently inhibited osteogenic differentiation, which was indicated by the downregulated osteogenic marker genes osterix (Osx), osteocalcin (OCN), and ALP both protein and mRNA levels after OM treatment (Fig. 2b, c). In addition, KLF2 mRNA and protein expression were found to be significantly downregulated after SNHG1 overexpression with or without OM treatment (Fig. 2b, c). Furthermore, upregulation of SNHG1 suppressed OM induced ALP activity and the matrix mineralization level (Fig. 2d, e), which confirmed that SNHG1 plays a negative role in the regulation of the osteogenic differentiation of PDLSCs.

### Downregulation of KLF2 inhibited osteogenic differentiation of PDLSCs

**Fig. 3:**
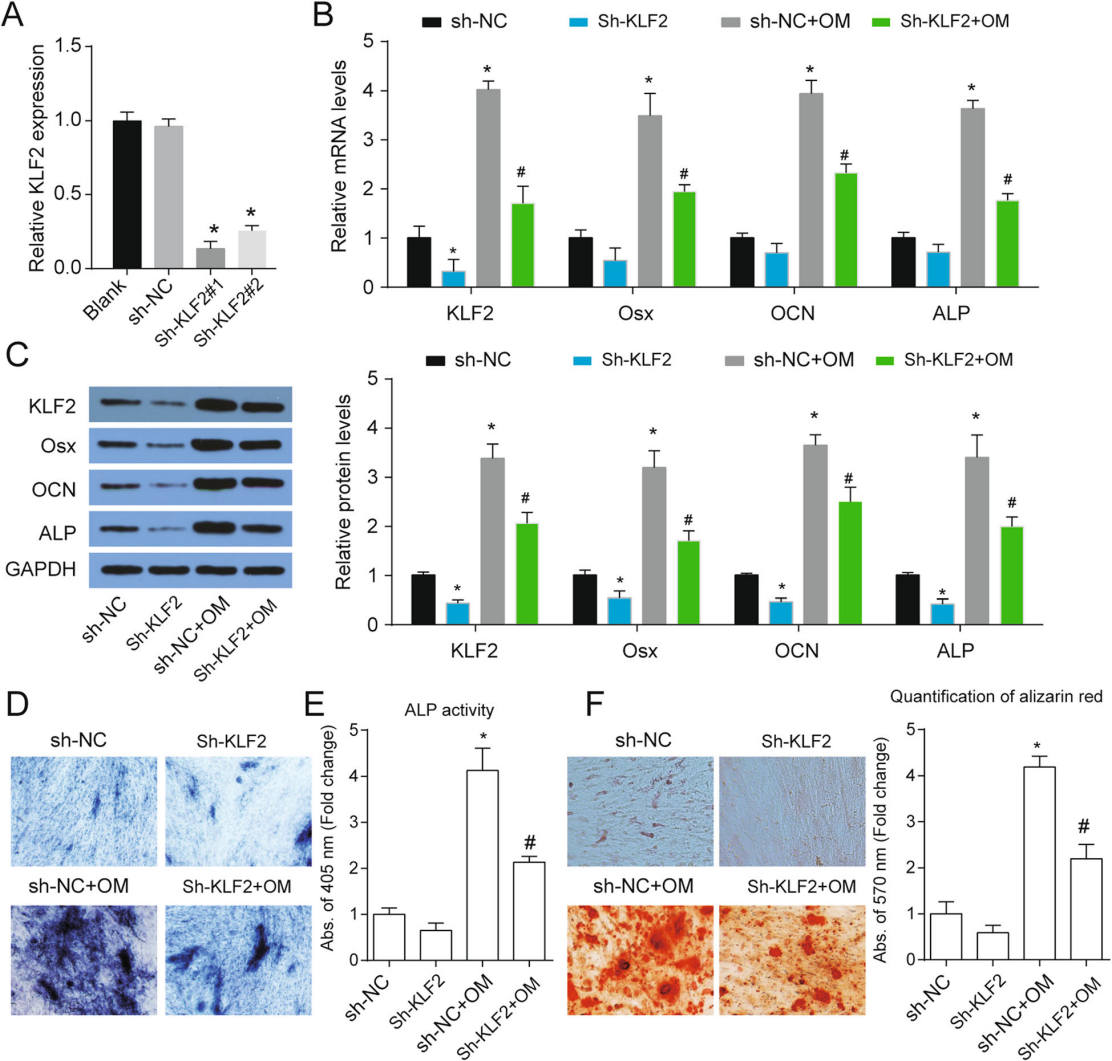
KLF2 knockdown inhibited osteogenic differentiation of PDLSCs. **a** The mRNA expressions of KLF2 in PDLSCs after treated with specific shRNAs of KLF2 for 48 h. **b** The mRNA expressions of KLF2 and osteoblastic marker genes Osx, OCN, and ALP were analyzed at day 14 of osteogenic differentiation after indicated treatment with GAPDH as a control. **c** The protein levels KLF2 and osteoblastic marker genes Osx, OCN, and ALP were analyzed at day 14 of osteogenic differentiation after indicated treatment with GAPDH as a control. **d** ALP staining of PDLSCs at day 14 of osteogenic differentiation after indicated treatment. **e** ALP activity was measured at day 14 of osteogenic differentiation after indicated treatment. **f** Alizarin Red staining of PDLSCs at day 14 of osteogenic differentiation after indicated treatment and quantification was shown at right. *p < 0.05 versus Sh-NC; ^#^*p* < 0.05 versus sh-NC+OM

To explore the role of KLF2 on PDLSCs osteogenic differentiation, specific shRNAs were employed to reduce KLF2 expression and sh-KLF2#1 was selected to further study for its higher efficiency (Fig. 3a). KLF2 knockdown apparently inhibited Osx, OCN, and ALP expression after OM treatment (Fig. 3b, c). ALP staining and ALP activity assays suggested that KLF2 knockdown inhibited ALP activity which was notably enhanced after OM treatment (Fig. 3d, e). In addition, Alizarin Red staining confirmed the inhibitory effect of sh-KLF2 on the osteoblast phenotype with the decrease of matrix mineralization level after sh-KLF2 treatment (Fig. 3f). Taken together, these results indicated that KLF2 plays a positive role in the regulation of the osteogenic differentiation of PDLSCs.

### KLF2 mediates SNHG1-regulated osteogenic differentiation of PDLSCs

**Fig. 4:**
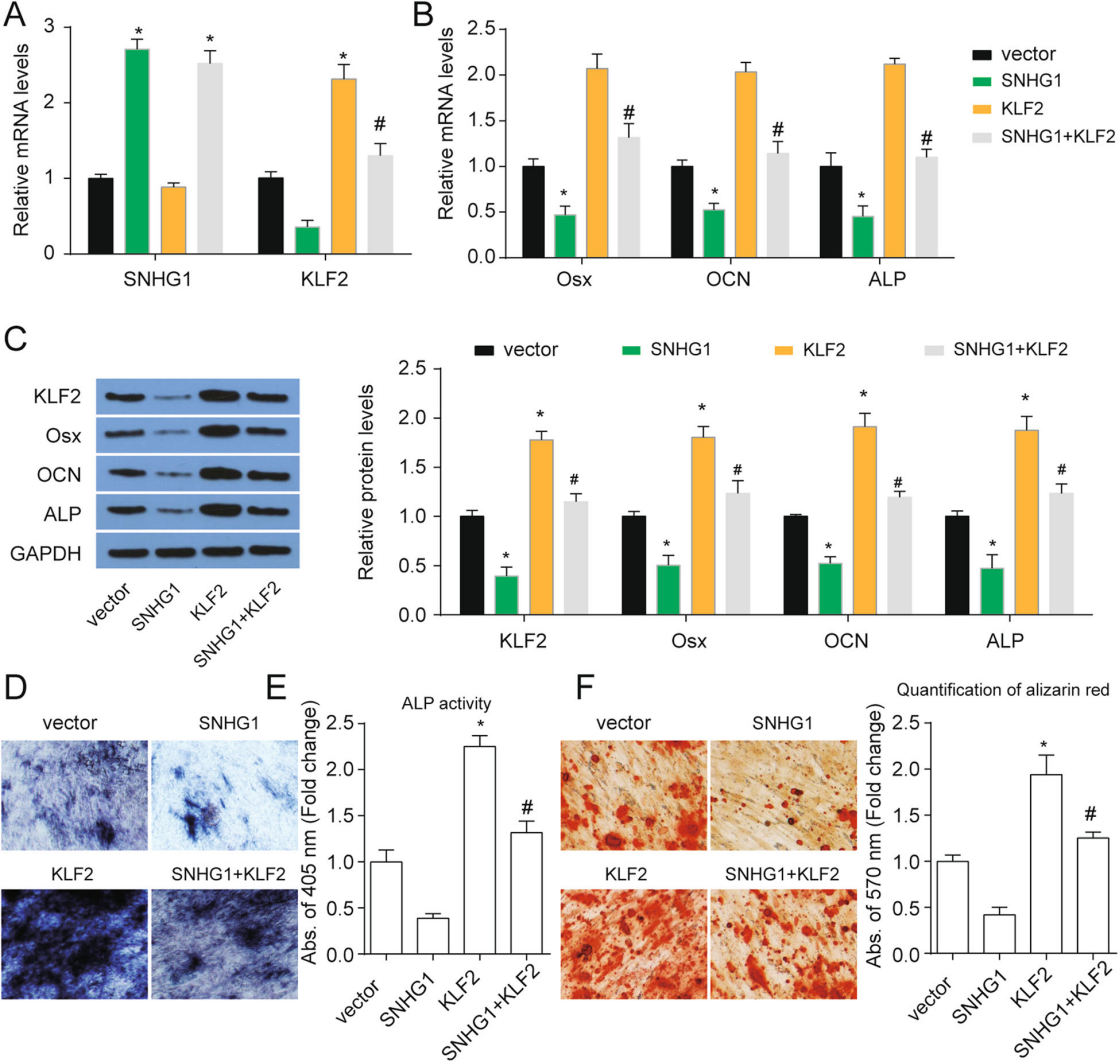
KLF2 mediates SNHG1-regulated osteogenic differentiation of PDLSCs. **a** The mRNA expressions of SNHG1 and KLF2 were analyzed at day 14 of osteogenic differentiation after indicated treatment with GAPDH as a control. **b** The mRNA expressions of osteoblastic marker genes Osx, OCN, and ALP were analyzed at day 14 of osteogenic differentiation after indicated treatment with GAPDH as a control. **c** The protein levels KLF2 and osteoblastic marker genes Osx, OCN, and ALP were analyzed at day 14 of osteogenic differentiation after indicated treatment with GAPDH as a control. **d** ALP staining of PDLSCs at day 14 of osteogenic differentiation after indicated treatment. **e** ALP activity was measured at day 14 of osteogenic differentiation after indicated treatment. **f** Alizarin Red staining of PDLSCs at day 14 of osteogenic differentiation after indicated treatment and quantification was shown at right. *p < 0.05 versus vector; ^#^*p* < 0.05 versus SNHG1

To ascertain the exact role of KLF2 in SNHG1-regulated osteogenic differentiation, PDLSCs were infected with SNHG1-lentivirus or KLF2-lentivirus. As shown in Fig. 4a, SNHG1 inhibited KLF2 expression and overexpression of KLF2 recovered KLF2 expression decreased by SNHG1. KLF2 overexpression did not affect SNHG1 levels. What is more, SNHG1 overexpression inhibited osteogenic differentiation of PDLSCs as the downregulated of osteogenic marker genes Osx, OCN, and ALP and decrease of ALP activity and the matrix mineralization level (Fig. 4b–f). KLF2 overexpression promoted osteogenic differentiation of PDLSCs and KLF2 overexpression impaired the effect of SNHG1 in the osteogenic differentiation of PDLSCs (Fig. 4b–f). These results indicated that KLF2 was involved in SNHG1-regulated osteogenic differentiation of PDLSCs.

### SNHG1 participates in epigenetic repression of KLF2 by interacting with EZH2

**Fig. 5:**
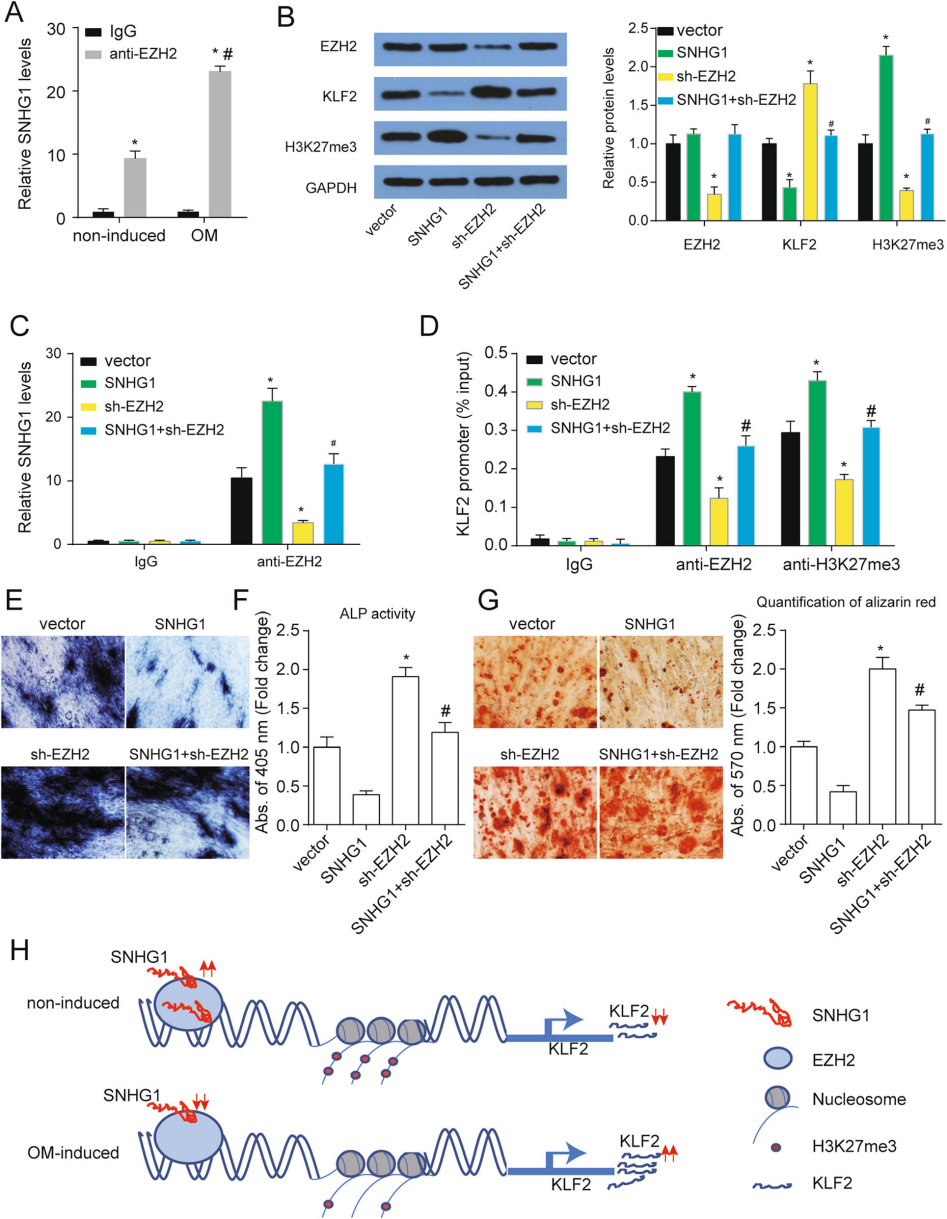
SNHG1 binds with EZH2 leading to epigenetically silencing of KLF2. **a** RIPs experiments for EZH2 were performed and the coprecipitated RNA was subjected to qRT-PCR for SNHG1 in PDLSCs with or without OM treatment. *p < 0.05 versus IgG; ^#^*p* < 0.05 versus non-induced. **b** The protein levels EZH2, KLF2, and H3K27me3 were analyzed at day 14 of osteogenic differentiation after indicated treatment with GAPDH as a control. **c** RIPs experiments for EZH2 were performed and the coprecipitated RNA was subjected to qRT-PCR for SNHG1 in PDLSCs after indicated treatment along with OM treatment. **d** ChIP assays were performed to detect EZH2 and H3K27me3 occupancy in the KLF2 promoter region. **e** ALP staining of PDLSCs at day 14 of osteogenic differentiation after indicated treatment. **f** ALP activity was measured at day 14 of osteogenic differentiation after indicated treatment. **g** Alizarin Red staining of PDLSCs at day 14 of osteogenic differentiation after indicated treatment and quantification was shown at right. **h** Proposed model of SNHG1 regulating KLF2 expression to inhibit osteogenic differentiation of PDLSCs. *p < 0.05 versus vector; ^#^*p* < 0.05 versus SNHG1

There are reports that a number of lncRNAs have been validated for working through the co-operation with chromatin-modifying enzymes and then accelerate epigenetic activation and silence the target gene expression [25]. As evident from Fig. 5a, amplification of endogenous SNHG1 was observed in the anti-EZH2 RNA immunoprecipitation fraction associated with the input, when compared with the IgG fraction in the PDLSCs with or without OM treatment. Importantly, the levels of endogenous SNHG1 was higher in osteogenic PDLSCs compared with non-induced PDLSCs. SNHG1 overexpression increased H3K27me3 protein levels suggesting that the total methylation levels of PDLSCs were enhanced after SNHG1 overexpression (Fig. 5b). We also found that SNHG1 overexpression did not affect EZH2 expression. Downregulation of EZH2 obviously inhibited EZH2 and H3K27me3 protein levels. Sh-EZH2 rescued EZH2 and H3K27me3 protein levels enhanced by SNHG1 overexpression (Fig. 5b). In addition, Downregulation of EZH2 promoted SLF2 expression and rescued SLF2 protein level inhibited by SNHG1 (Fig. 5b). SNHG1 overexpression enhanced the enrichment of SNHG1 in product pulled-down by EZHE antibody and sh-EZH2 treatment decreased the enrichment of SNHG1 in product pulled-down by EZHE antibody (Fig. 5c). To further address whether SNHG1 was involved in transcriptional repression through enrichment of H3K27me3 to the promoter regions of KLF2, we conducted ChIP assays. We found that SNHG1 overexpression increased EZH2 binding ability to their promoters. Similarly, EZH2 enrichment induced H3K27me3 modifications also increased in their promoter regions after SNHG1 overexpression (Fig. 5d). Down-expression of EZH2 rescued the enrichment of EZH2 and H3K27me3 on the promoter regions of KLF2 induced by SNHG1. SNHG1 knockdown notably inhibited SNHG1 expression and promoted KLF2 expression, whereas there was no significant change for EZH2 expression (Figure S1A). In addition, SNHG1 knockdown reduced the enrichment for EZH2 and H3K27me3 on KLF2 promoter region (Figure S1B). Furthermore, EZH2 knockdown promoted osteogenic differentiation of PDLSCs and rescued the effect of SNHG1 in the osteogenic differentiation of PDLSCs (Fig. 5e–g). Taken together, these results indicated that SNHG1 epigenetically silenced KLF2 transcription through EZH2-mediated H3K27me3 methylation in the progress osteogenic differentiation of PDLSCs (Fig. 5h).

### SNHG1/EZH2/KLF2 axis promoted PDLSC proliferation

As cell proliferation has a strong influence on cell differentiation [26], we further explored the effect of SNHG1/EZH2/KLF2 axis on PDLSC proliferation. SNHG1 overexpression promoted PDLSC proliferation (Figure S1A). KLF2 overexpression inhibited PDLSC proliferation, whereas KLF2 downregulation promoted PDLSC proliferation (Figure S1B-C). EZH2 downregulation inhibited PDLSC proliferation (Figure S1D). KLF2 overexpression or EZH2 downregulation partly reversed the promotion of SNHG1 on PDLSC proliferation (Figure S1C-D). In short SNHG1/EZH2/KLF2 axis promoted PDLSC proliferation.

## Discussion

Osteogenic differentiation is one of the most crucial characteristics of the oral stem cells pluripotency. It plays an important role in periodontal tissue regeneration and engineering. However, the regulatory mechanisms of MSC fate determination remain poorly understood. In this study, we found that epigenetic silencing of KLF2 by lncRNA SNHG1 inhibits PDLSC osteogenesis differentiation.

LncRNAs were reported to be involved in many biological processes, such as transcription, post-transcription, and translational regulation of gene expression [27]. LncRNA may act as a competing endogenous (ceRNA) for miRNAs, adjusting the expression of their targeting genes in the osteogenic differentiation of MSC [28, 29]. Recently reports have also demonstrated that lncRNA could serve as epigenetic regulators participating in the osteogenic differentiation of MSC. For example, lncRNA MEG3 inhibited osteogenic differentiation of human dental follicle stem cells by epigenetically regulating wnt pathway [30]. HoxA-AS3 was reported to act as an epigenetic switch through binding with EZH2 and inhibit the transcription of key osteoblastic factors in bone marrow mesenchymal stem cells [31]. HOTAIRM1 promoted osteogenesis of dental follicle stem cells by epigenetically regulating HOXA2 [32]. In our study, we found that SNHG1 epigenetically silenced KLF2 transcription through EZH2-mediated H3K27me3 methylation in the progress osteogenic differentiation of PDLSCs.

EZH2 has been reported to be involved in various biological processes including cell growth and differentiation. Recent studies unraveled an importance role of EZH2 in regulating osteogenic differentiation [33, 34]. EZH2 induced Histone H3 lysine 27 (H3K27) methylation resulting in transcriptional repression of osteoblastic factors [31]. However, the underlying mechanisms of the impact of EZH2 on PDLSCs osteogenic differentiation still need to be explored. In this study, we found the binding of EZH2 and SNHG1 enhanced in the osteogenesis of PDLSCs. Furthermore, ChIP-PCR analysis showed that inhibition of EZH2 depressed KLF2 transcription. We confirmed that SNHG1/EZH2 regulates the osteogenesis of PDLSCs through epigenetically mediating KLF2 expression.

Krüppel-like factor 2 (KLF2) is a zinc finger structure and DNA-binding transcription factor and is reported to possesses multiple biological functions as cellular growth and differentiation. It has been reported that KLF2 regulates cellular growth and differentiation [35]. KLF2 was involved in degradation of type II collagen, a primary component of the extracellular matrix in articular cartilage, through inhibiting matrix metalloproteinase-13 expression [36]. Interestingly, KLF2 was reported to be significantly increased during the osteoblastic differentiation process and KLF2 could promote the expression of the osteoblastic differentiation marker genes Alp, Osx, and Ocn and stimulate mineralization by enhancing the expression of and interacting with Runx2 [37]. In our study, we also found that the expression of KLF2 was upregulated during the osteoblastic differentiation process of PDLSCs and KLF2 knockdown could inhibit the expression of the osteoblastic differentiation marker genes Alp, Osx, and Ocn and stimulate mineralization.

## Conclusions

SNHG1 inhibited the osteogenic differentiation of PDLSCs through EZH2-mediated H3K27me3 methylation of KLF2 promotor. Our results provide new insight indicating that SNHG1-KLF2 axis possesses great potential as a novel class of therapeutic targets for bone regeneration.

## Supplementary information

**Additional file 1: Supplementary table 1.** The sequences of shRNA.

**Additional file 2: Supplementary Table 2.** The primers used in this manuscript.

**Additional file 3: Supplementary Table 3.** The antibody used in this manuscript.

**Additional file 4:** **Supplementary Figure 1.** (A) The expression of SNHG1, EZH2 and KLF2 after SNHG1 knockdown. (B) The enrichment for EZH2 and H3K27me3 on KLF2 promoter region after SNHG1 knockdown. *p < 0.05 vs. sh-NC. (C) The cell viability of PDLSCs after SNHG1 overexpression. **p < 0.01 vs. vector. (D) The cell viability of PDLSCs after KLF2 knockdown. **p < 0.01 vs. sh-NC. (E) The cell viability of PDLSCs after altering SNHG1 or KLF2 expression. **p < 0.01 vs. vector. (F) The cell viability of PDLSCs after altering SNHG1 or EZH2 expression. *p < 0.05 vs. vector.

## Data Availability

The dataset(s) supporting the conclusions of this article is (are) included within the article.

## Abbreviations

ANOVA: Analysis of variance
ChIP: Chromatin immunoprecipitation
ECL: Enhanced chemiluminescence
EZH2: Enhancer of the zeste homolog 2
H3K27: Histone H3 lysine 27
KLF2: Kruppel-like factor 2
lncRNAs: Long noncoding RNAs
OM: Osteogenic differentiation medium
PDL: Periodontal ligament
PDLSCs: Periodontal ligament stem cells
