# Supplementary material for: Epigenetic silencing of KLF2 by long non-coding RNA SNHG1 inhibits periodontal ligament stem cell osteogenesis differentiation
Source: Stem Cell Res Ther. 2020 Oct 7;11:435. doi: 10.1186/s13287-020-01953-8 (PMC7539403; doi:10.1186/s13287-020-01953-8)
Supplement: Supplementary file 1 — Additional file 1: Supplementary table 1. The sequences of shRNA. [file 13287_2020_1953_MOESM1_ESM.docx]

**Supplementary table 1** **The sequences of shRNA.**

| Gene title | Oligonucleotides (5’-3’) |
| --- | --- |
| Sh-EZH2  forward | CACCGCAAATTCTCGGTGTCAAATTCGAAAATTTGACACCGAGAATTTGC |
| Sh-EZH2  reverse | AAAAGCAAATTCTCGGTGTCAAATTTTCGAATTTGACACCGAGAATTTGC |
| Sh-KLF2#1  forward | CACCGCAAGACCTACACCAAGAGTTCGAAAACTCTTGGTGTAGGTCTTGC |
| Sh-KLF2#1  reverse | AAAAGCAAGACCTACACCAAGAGTTTTCGAACTCTTGGTGTAGGTCTTGC |
| Sh-KLF2#2  forward | CACCGCTGCACATGAAACGGCACATCGAAATGTGCCGTTTCATGTGCAGC |
| Sh-KLF2#2  reverse | AAAAGCTGCACATGAAACGGCACATTTCGATGTGCCGTTTCATGTGCAGC |
| Sh-SNHG1#1  forward | CACCGCTTAAAGTGTTAGCAGACATTCGAAAATGTCTGCTAACACTTTAAG |
| Sh-SNHG1#1  reverse | AAAACTTAAAGTGTTAGCAGACATTTTCGAATGTCTGCTAACACTTTAAGC |
| Sh-SNHG1#2  forward | CACCGTGTATCTAAAAAACAAAAGGGCGAACCCTTTTGTTTTTTAGATACA |
| Sh-SNHG1#2  reverse | AAAATGTATCTAAAAAACAAAAGGGTTCGCCCTTTTGTTTTTTAGATACAC |
| Sh-NC  forward | CACCGTTCTCCGAACGTGTCACGTTTCGAAAAACGTGACACGTTCGGAGAA |
| Sh-NC  reverse | AAAATTCTCCGAACGTGTCACGTTTTTCGAAACGTGACACGTTCGGAGAAC |
