# Supplementary material for: Epigenetic silencing of KLF2 by long non-coding RNA SNHG1 inhibits periodontal ligament stem cell osteogenesis differentiation
Source: Stem Cell Res Ther. 2020 Oct 7;11:435. doi: 10.1186/s13287-020-01953-8 (PMC7539403; doi:10.1186/s13287-020-01953-8)
Supplement: Supplementary file 2 — Additional file 2: Supplementary Table 2. The primers used in this manuscript. [file 13287_2020_1953_MOESM2_ESM.docx]

**Supplementary Table 2. The primers used in this manuscript.**

| **GENE** | **Forward primer** | **Reverse primer** |
| --- | --- | --- |
| SNHG1 | ACAGCAGTTGAGGGTTTGCT | GGGCCTGGATCATGTAAGAA |
| EZH2 | TGCACATCCTGACTTCTGTG | AAGGGCATTCACCAACTCC |
| KLF2 | CTGCACATGAAACGGCACAT | CAGTCACAGTTTGGGAGGGG |
| *OSX* | CTCCTTTCACCTGCAGGCAG | CAGACAGTCAGAAGAGCTGT |
| OCN | ACACTCCTCGCCCTATTG | GATGTGGTCAGCCAACTC |
| ALP | GAGATGGATGGCCAGTGCAAGC | GCTGTGTGCCAAGCCTTTCCC |
| U6 | CTCGCTTCGGCAGCACA | AACGCTTCACGAATTTGCGT |
| GAPDH | AGCCACATCGCTCAGACAC | GCCCAATACGACCAAATCC |
