# Supplementary material for: Epigenetic silencing of KLF2 by long non-coding RNA SNHG1 inhibits periodontal ligament stem cell osteogenesis differentiation
Source: Stem Cell Res Ther. 2020 Oct 7;11:435. doi: 10.1186/s13287-020-01953-8 (PMC7539403; doi:10.1186/s13287-020-01953-8)
Supplement: Supplementary file 3 — Additional file 3: Supplementary Table 3. The antibody used in this manuscript. [file 13287_2020_1953_MOESM3_ESM.docx]

**Supplementary Table 3. The antibody used in this manuscript.**

| **Antibody** | **Product code** | **Supplier** |
| --- | --- | --- |
| KLF2 | ab236507 | Abcam |
| Osx | ab229258 | Abcam |
| OCN | ab13420 | Abcam |
| ALP | ab228636 | Abcam |
| EZH2 | ab228697, ChIP grade | Abcam |
| H3K27me3 | ab272155, ChIP grade | Abcam |
| Ago2 | ab32381, ChIP grade | Abcam |
| GAPDH | ab181602 | Abcam |
