# Supplementary figures and images for: Epigenetic silencing of KLF2 by long non-coding RNA SNHG1 inhibits periodontal ligament stem cell osteogenesis differentiation
Source: Stem Cell Res Ther. 2020 Oct 7;11:435. doi: 10.1186/s13287-020-01953-8 (PMC7539403; doi:10.1186/s13287-020-01953-8)

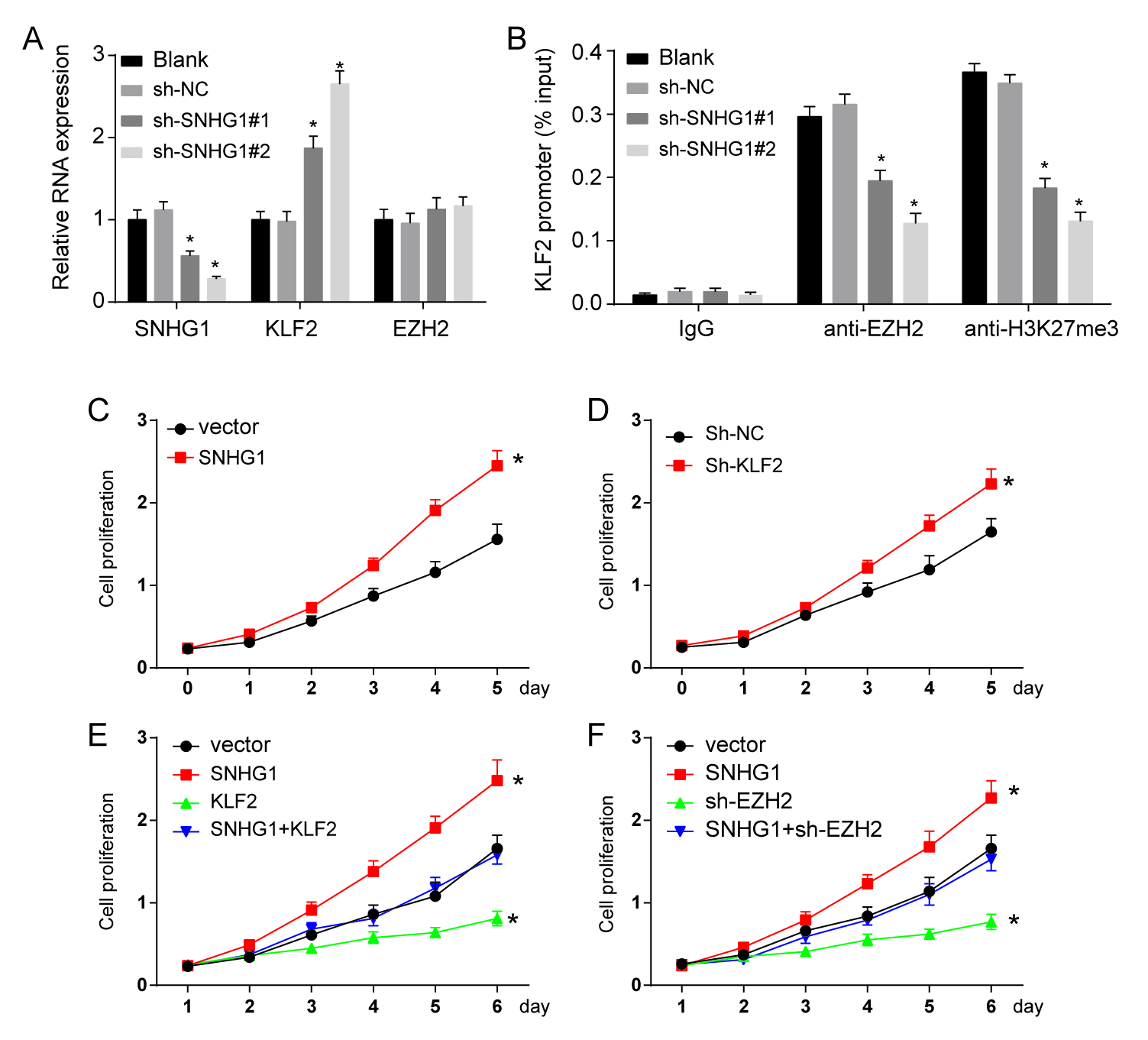

Supplement: Supplementary file 4 — Additional file 4: Supplementary Figure 1. (A) The expression of SNHG1, EZH2 and KLF2 after SNHG1 knockdown. (B) The enrichment for EZH2 and H3K27me3 on KLF2 promoter region after SNHG1 knockdown. *p < 0.05 vs. sh-NC. (C) The cell viability of PDLSCs after SNHG1 overexpression. **p < 0.01 vs. vector. (D) The cell viability of PDLSCs after KLF2 knockdown. **p < 0.01 vs. sh-NC. (E) The cell viability of PDLSCs after altering SNHG1 or KLF2 expression. **p < 0.01 vs. vector. (F) The cell viability of PDLSCs after altering SNHG1 or EZH2 expression. *p < 0.05 vs. vector. [file 13287_2020_1953_MOESM4_ESM.tif]
